# Exploring the determinants of users’ co-creation behavior on music streaming platforms in China

**DOI:** 10.1371/journal.pone.0291313

**Published:** 2023-12-27

**Authors:** Jinghong Xu, Jiankun Gong, Dan Ji

**Affiliations:** 1 School of Journalism and Communication, Beijing Normal University, Beijing, China; 2 Department of Media and Communication Studies, Faculty of Arts and Social Sciences, University of Malaya, Wilayah Persekutuan Kuala Lumpur, Malaysia; 3 School of Humanity, Shanghai Jiao Tong University, Shanghai, China; 4 School of International and Public Affairs, China Institute for Urban Governance, Shanghai Jiao Tong University, Shanghai, China; COMSATS University Islamabad - Wah Campus, PAKISTAN

## Abstract

Music streaming platforms have recently become one of the latest innovative music devices used to replace traditional music sets. In order to examine users’ behavior on music streaming platforms, this study proposes an extended research model based on flow theory and investigates the relationship between flow experience and co-creation behavior. A partial least square methodology was employed to test the proposed model and corresponding hypotheses on data collected from 390 survey samples. The results showed that flow experience has a significant influence on users’ co-creation behavior. Among the three antecedents, only perceived skill and perceived interactivity have the strongest effects on flow experience, while perceived control has little effect on flow experience. This study discusses some valuable theoretical implications and offers insights useful for both researchers and practitioners.

## Introduction

In the past few years, new media technologies have made progress in digital content circulation, e.g., music streaming platforms [1]. The growing popularity of music streaming platforms has made online music a major culture industry in China. According to the Chinese Industrial Institution, the online audience in the music industry now cover more than 70% of internet users. The online music industry has been significantly affected by the rapid development of digital technologies [2, 3].

In the late 2010s, music streaming platforms, such as QQ, KuGou, and Netease opened new markets in the online music industry through improvements in networking and multimedia technology. Music streaming platforms have the potential to transform the social dynamics of music consumption in ways not formerly encountered [4]. It can not only have broadcasting functions but also application stores that enable users to access millions of music products at little or no cost and also make it possible for users from different backgrounds to approach licensed music at anytime and anywhere. What’s more, users can find music they like in the large variety of music offered by automatic tag recommendations on music streaming platforms, and can also have a chance to share their personal experiences of consuming music on these platforms.

The music streaming platforms generally bestows users with social elements through three sections including Dynamic, Group, and Squares. In the Dynamic section, everyone can see the music content such as lyrics, songs, and playlists their friends post in real-time, whose songs their friends listen to. Everyone can communicate with each other through the actions of Liking, Forwarding, and Remarking. The Group section is just like the SNS’s function of Group but has some little difference. It allows people to join the music stars’ groups, follow the music popular stars and check the dynamics of stars, find stars’ songs, lyrics they listen to, and their playlists, and communicate with stars directly. In the Squares section, users can post what they like in public and communicate with other strangers who have the same interests in the content.

By drawing on music automatic tag recommendations and those social elements, these platforms have the potential to gather music resources and create new value. The new platforms essentially alter the relationship between users and producers by supplying new forms of dialogue in the production-consumption field of the music industry.

New forms of online music production and distribution have gradually adapted to dynamic market demands, leading to greater investigation of customers’ behavior on music streaming platforms, especially the value co-creation behavior including creating and maintaining their playlists, posting their opinions, comments, and reviews of the lyrics on the platforms.

Most studies in the online music environment have explored the field of music listening behavior, but few focused on the motivation of the audiences’ online music value co-creation behavior. As music platforms continue to proliferate, there is growing interest in identifying factors that influence how individuals collaborate and create new music content.

Online music content co-creation behavior primarily occurs when individuals are motivated to access the platform, listen to the music and look through the reviews posted, choose those they are interested in, are willing and able to follow, and take the time and effort to formulate and post their content related. Although online music content co-creation behavior may take on a variety of forms, the focus here is on two key aspects: the volume of music content contributed through the posting of response messages, and the average helpfulness of those responses directly deepens the others’ cognition of the music works.

As there are few business aspects and empirical studies focused on users’ behavior concerning music content co-creation, discussions as to the factors that affect the users’ co-creation behavior have not been adequately addressed. Users’ co-creation behavior is indeed complex [5]. The current emerging view is that personal experiences and the abilities to share their experiences with others are key to value creation in music production, for music works as cultural production focuses on the users’ experiences.

What is it that motivates users to expend considerable time and effort co-creating new content on music streaming platforms? Prior research has shown that flow theory is a useful construct to understand the impact of user cognition on computer-mediated technologies [6]. The flow theory was first proposed by Csikszentmihalyi [7] as a way of understanding motivation. Initially, researchers employed flow theory to explain the optimal state of athletes. And then flow theory has subsequently been applied in many other fields including sports, leisure, education, and so on [8–11]. More and more researchers study users’ flow experience in online behavior. Wang and Huang [12] proposed a conceptual research model in the context of music streaming services, using flow theory as a focal construct to examine its antecedents and the effect on users’ intentions to continue to subscribe to the service. Yang and Lee [13] recently studied the user acceptance of streaming media devices from flow theory. However, their discussions are limited to that device acceptance while ignoring the users’ content co-creation behavior.

How does users’ flow experience affect their co-creation behavior? This paper contributes to investigating the motivation of the users’ co-creation behavior in music content creation online by using the flow theory. The primary objective of this study is to establish empirical models to test the theoretical construct of flow experience on music streaming platforms and discuss the relationship between flow experience and users’ co-creation behavior on music streaming platforms in China. The results of this study are of significance for both music producers and audiences.

## Literature review

This study aimed to contribute to the existing knowledge about factors that encourage individuals to engage in co-creation. The specific factor examined was the users’ flow experience. It hoped that by examining the relationship between users’ flow experience and initial user engagement in co-creation, the platforms could develop better strategies to encourage their customers to participate. Because participation in co-creation is increasing, the knowledge contributed by this study is of high value.

### Flow theory

The flow theory has been proposed as a useful framework for studying the experience of individuals when they are involved in doing something and for identifying the factors that influence this experience. The concept of flow means a peculiar dynamic state the holistic sensation that people feel when they act with total involvement and nothing else seems to matter [7]. Flow is a sensation present when individuals are involved in their actions. This state assumes no conscious intervention [14]. As an intrinsic motivation, flow refers to engaging in an activity without receiving any apparent reinforcement, such as simply for enjoyment [11]. When people experience flow, they are immersed in their activity, and current actions transit flawlessly into another, displaying an inner logic of their own and creating harmony. The user experiences a seamless transition and total control of the actions without interruption and the user becomes absorbed in the activity. Lately, the flow has been thought as a critical construct to understand consumer behavior in online environments and online customer experiences [15].

According to Csikszentmihalyi, flow is a personal aspect of the generation of creative ideas and products, and some research supports the relationship between flow and creativity, flow and value creation [16, 17], flow is more likely to occur and is more ardent and complex in structured activities, such as the arts, sports, and certain occupations [18].

The experience itself is so enjoyable that people will do it even at a great cost, for the sheer sake of doing it [19]. The subjective experience is generally regarded as a function of three variables: perceived control, perceived skill, and perceived interactivity.

### Perceived control

Perceived control is defined as the users’ sense of the level that one can control over the environment and one’s intended actions [20]. In this current research, perceived control is defined as the level of a user’s control over content production on music streaming platforms.

In the extant literature, perceived control was put in different positions in flow structure in prior research [21]. Perceived control was one of the factors affecting the experience of flow [22]. Novak [23] regarded perceived control as the consequence of flow. Trevino and Webster [24] considered perceived control to be one of the core flow constructs. Zaman and Anandarajan [25] discussed that flow is positively affected by users’ perceived control in the usage of instant messaging services. Lee and Chiang [26] also found that the players’ flow state is enhanced when they feel a high level of control over their actions within the game.

In this study, we thought that one of the reasons why people enjoy participating in co-creation is the powerful sense of control. For the users who use the platform, the environment is quite different from traditional face-to-face communication, and the users demand more control over the content creation on the platform. So in our study, perceived control is viewed as one of the determinants of the flow experience in a platform environment. The first hypothesis is:

**H1: Perceived control is positively related to flow experience on music streaming platforms**.

### Perceived skill

Perceived skill is defined as an individual’s judgment of his/her capabilities for using his/her knowledge or performing specific tasks [27]. This definition is similar to self-efficacy, indicating or revealing how the individual trusts his/her abilities and skills to face challenges [28]. In this study, perceived skill is defined as a user’s subjective judgment of his/her capability to co-create content on music streaming platforms.

Research has found that the level of an individual’s skills is one of the most important antecedents of flow [22]. Zimmerman demonstrated that individuals’ beliefs about capabilities are essential in learning course and increases the motivation to study [29]. Perceived skill is important because people with a high level of perceived skills are more likely to make an effort to face their environment and immerse in the task when facing possible negative outcome expectations. Therefore, the second hypothesis is:

**H2: Perceived skill is positively related to flow experience on music streaming platforms**.

### Perceived interactivity

Digital technologies such as platforms have enabled users to play a more active role than before in all their activities including interaction [30]. Music streaming platforms allow hundreds of users to interact with others when they listen to the same song. Interactivity is one of the most important characteristics of human-computer interaction.

Trevino and Webster [24] described the interaction between humans and computers and explained such interaction as being an experience of enjoyment and exploration. Novak [23] considered that interactivity in Web navigation can be made by three dimensions, namely, speed, mapping, and range. Mapping of interaction indicates a perceived natural and intuitive interaction. Speed and range of interaction refer to the number of possible actions in a given time.

Massey and Levy [31] noted that the Web provides for "interpersonal interactivity" because people can communicate with one another through tools such as chat rooms and bulletin boards. By making their Web sites friendly to users, marketers can facilitate this kind of interpersonal interactivity and generate positive word of mouth for their companies [32]. Xiang and Chae [33] investigated five dimensions of perceived interactivity positively related to satisfaction and belonging and even further affect the continuance intentions in the context of the video-sharing platform.

Interpersonal communication through music streaming platforms also can facilitate users’ intrinsic enjoyment and interest in interaction. Users get a sense of having fun or optimal experience in interactivity. Thus, the third hypothesis is:

**H3: Perceived interactivity is positively related to flow experience on music streaming platforms**.

### Co-creation

The literature associated with co-production and co-creation has been reviewed by many researchers [34–36]. Co-creation refers to constructive participation in creation, including the ability to share information and cooperate. Central to service-dominant (S-D) logic is the proposition that the customer becomes a co-creator of value [37]. presented that more and more consumers themselves increasingly participate in creating cultural products. The ability of consumers’ co-creation is affected by technological change and driven by consumers’ preferences to participate in the creative process of making and remaking cultural goods—is a new phenomenon that has a significant effect on businesses, consumers, and culture in general [38].

Previous research has focused on the positive consequences of flow. Users’ flow experienced during an activity online may lead to a positive behavioral outcome such as increased purchase intention, a cooperative act of joy and satisfaction, trust, and brand loyalty [25, 39–41]. Thus, the fourth hypothesis is:

**H4: Flow experience is positively related to users’ co-creation behavior on music streaming platforms**.

**Fig 1:**
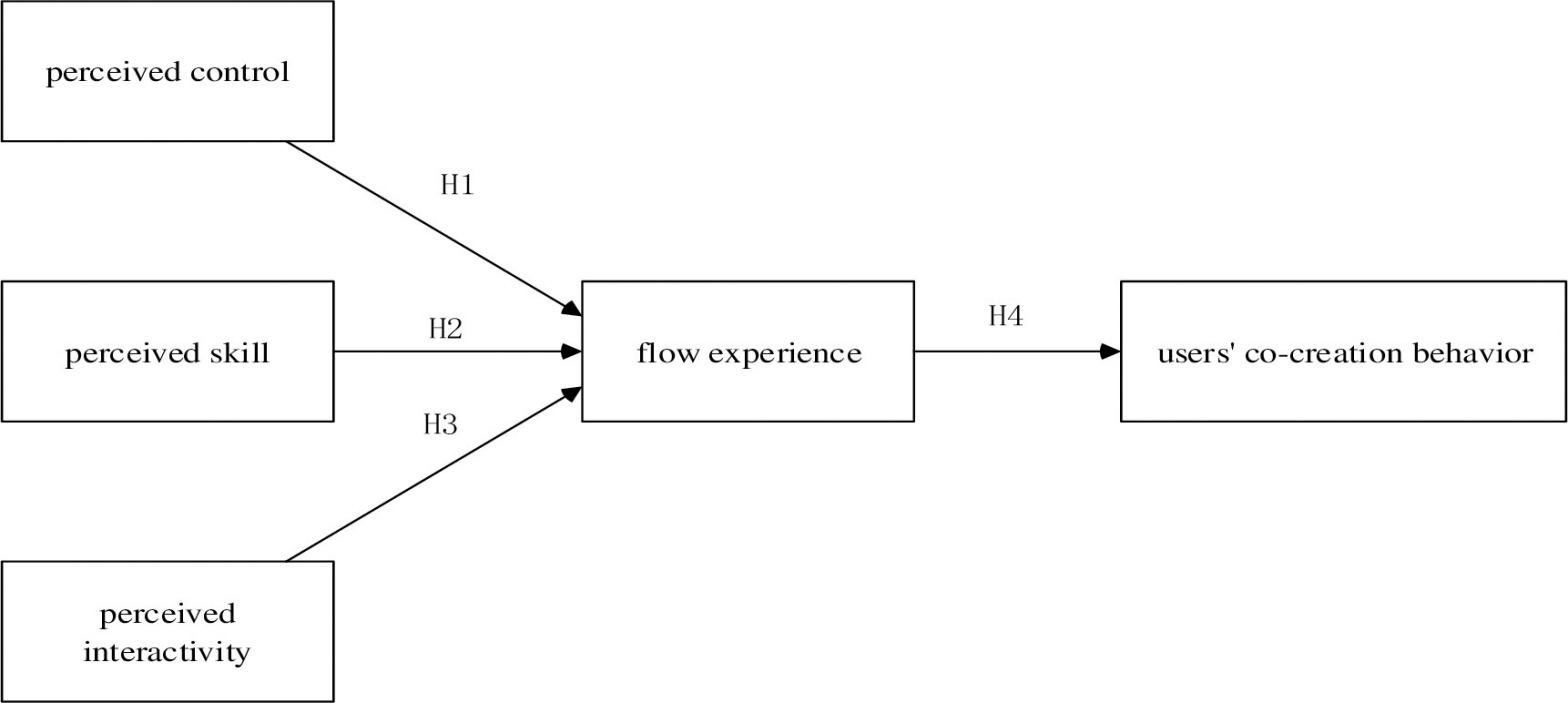
Research model.

**Fig 1** summarizes the structural equation model presenting, the proposed relationships of the determinants of flow experience toward users’ co-creation behavior. The proposed antecedents to flow experience on music streaming platforms include perceived control, perceived skill, and perceived interactivity.

## Research methodology

To test the research model, a survey was conducted. Data gathered from the survey were analyzed by SPSS and Smart PLS 3.0.

By carefully following the IRB protocol approved by the university where data collection was conducted in Oct 2021 (IRB202071). The questionnaire was posted to Wenjuanxing (https://www.wjx.cn/), a specialized survey distribution platform with 33.24 million users in China. We distributed the questionnaire through social network software, like chat groups of music lovers on social media, to the potential respondents.

### Data collection

The survey items were developed and adopted by the existing measures. The instrument was then reviewed and revised by two professors in communication and management to enhance the face and content validity. To avoid misunderstanding the meaning of the instrument, the questionnaire was distributed to 5 experts on social media and slightly revised according to their feedback. 20 graduate students in the Department of cultural industry study at Shanghai Jiao Tong University were pilot-tested, and all constructs were found to be valid and reliable.

The samples of this article are people who use the online music platform on both PC and mobile devices. We used the screening conditions that participants had to be music streaming platform users in the study. To encourage participation, we offered all respondents to take part in a lottery to get 10 RMB. We collected an overall gross sample consisting of 400 observations, which is a final sample of 390 valid responses for subsequent analysis after deleting unusable ones. Of the 390 respondents, 35.13 percent were male, and 64.87 percent were female. For age distribution, 0.51 percent of the participants were aged under 17, 45.13 percent were aged between 18 and 25, 31.54 percent were aged between 26 and 35, 17.69 percent were aged between 36 and 45, and 5.13 percent were aged above 46. Regarding the education of the respondents, 41.03 percent were graduates and 58.97 percent were undergraduates. The basic statistics are shown below (Table 1).

**Table 1:** Demographic information of respondents.

Demographic variables	Item	Frequency	Percentage
Gender	male	137	35.13
	female	253	64.87
Age	≤17	2	0.51
	18–25	176	45.13
	26–35	123	31.54
	36–45	69	17.69
	≥46	20	5.13
Education	graduate students	160	41.03
	undergraduate students	230	58.97
Number of responses	390		

### Measures

The questionnaire was first designed based on previous studies. All items were measured with five-point Likert scales, ranging from strongly disagree through neutral to strongly agree [42].

18 items for 5 constructs were obtained. Perceived control was measured by three items (PC1-3), perceived skill by three items (PS1-3), perceived interactivity was tested by three items (PI1-3), flow experience was explained by four items (FL1-4), and co-creation behavior was measured by five items (CC1-5). The specific contents of the questionnaire are shown below (Table 2).

**Table 2:** Survey items.

Items		
**Perceived Control**		
PC1 PC2 PC3	I feel I have the ability to influence the popularity of music on this music streaming platform.	[22]
	I think I can influence the expression of music connotations on this music streaming platform.	
	I think I can change the way other people think about this music on this music streaming platform.	
**Perceived Skill**		
PS1 PS2 PS3	I have relevant knowledge of music on this music streaming platform.	[23]
	I can understand the music on this music streaming platform.	
	I understand some techniques and methods of music on this music streaming platform.	
**Perceived Interactivity**		
PI1 PI2 PI3	This music streaming platform allows me to have a conversation with several listeners with similar interests.	[43]
	This music streaming platform allows me to resonate with a large number of listeners with similar interests.	
	This music streaming platform allows me to share some ideas with several listeners with similar interests.	
**Flow Experience**		
FL1 FL2 FL3 FL4	I think this music streaming platform can keep me happy.	[22]
	I have a lot of fun using this music streaming platform.	
	This music streaming platform has brought me a beautiful and unforgettable experience.	
	Using this music streaming platform can make me enjoy myself and forget the troubles in reality for the time being	
**Co-creation Behavior**		
CC1 CC2 CC3 CC4 CC5	I would like to create playlists with other listeners on this music streaming platform.	[44]
	I would like to contribute my professional knowledge to this music streaming platform for the benefit of other listeners.	
	I would like to provide information on this music streaming platform for other players to search for or answer questions.	
	When I participate in the creation of music reviews or playlists on this music streaming platform, I will prepare some relevant materials in advance.	
	When I have questions about music content on this music streaming platform, I will ask other listeners.	

All measurement items were adapted from prior studies. Since some items were modified for fitting into the research context, the reliability and validity of these items were checked by applying Cronbach’s alpha test and confirmatory factor analysis. Eighteen measurement items describe five latent constructs: flow experience, perceived control, perceived skill, perceived interactivity, and co-creation behavior.

## Data analysis and results

### Common method bias

Before the PLS assessment, a priori and post hoc procedures were taken to address common method bias [45, 46]. The procedural remedies comprised piloting the survey instrument including both the content and presentation to ease the respondents. CMV occurs when all the variables in a study are examined using the same instrument. Harman’s single-factor test in SPSS produced a result of 27% as the maximum variance explained by a single factor, which was lower than the threshold of 50% [47]. Therefore, CMV was not an issue in this study.

Following the recommendations by Hair Jr and Matthews [48], data was analyzed and interpreted in two stages, namely the assessment of the measurement model, and the assessment of the structural model.

### Measurement model

This study employed the structural equation modeling (SEM) analysis to evaluate the quality of the measurement tool and test the research hypotheses through the partial least squares (PLS) methodology with Smart PLS 3.0. PLS is appropriate given the sample size (n = 390), the focus on each path coefficient, and the focus on variance explained rather than overall model fit [49].

Reliability, convergent validity, and discriminant validity were tested to confirm construct reliability and validity. Reliability refers to the internal consistency of the scales, which can be determined through Cronbach’s alpha. A Cronbach’s alpha value in the excess of 0.70 can be regarded as statistically reliable[48]. Table 3 shows that Cronbach’s alphas for all eight constructs are also above the recommended level of reliability.

**Table 3:** Assessment of construct reliability and convergent validity.

	Items	Factor loading	Std. error	t value	AVE (>0.5)	Composite reliability (>0.6)	Cronbach’s alpha (>0.7)
Perceived control	PC1	0.963	0.027	13.537	0.857	0.947	0.916
	PC2	0.910	0.035	9.483			
	PC3	0.903	0.036	10.096			
Perceived skill	PS1	0.884	0.023	15.522	0.767	0.908	0.848
	PS2	0.881	0.031	13.366			
	PS3	0.861	0.029	12.217			
Perceived interactivity	PI1	0.857	0.066	7.624	0.608	0.821	0.673
	PI2	0.638	0.091	3.662			
	PI3	0.825	0.048	8.756			
Flow experience	FL1	0.756	0.032	8.755	0.597	0.855	0.776
	FL2	0.766	0.033	9.329			
	FL3	0.763	0.049	6.849			
	FL4	0.804	0.045	7.713			
Co-creation	CC1	0.824	0.043	6.404	0.641	0.899	0.860
	CC2	0.820	0.027	8.685			
	CC3	0.775	0.034	7.729			
	CC4	0.769	0.039	6.029			
	CC5	0.813	0.035	6.475			

Convergent validity is the degree to which multiple items adopted to measure the same construct. Factor loadings, composite reliability (CR), and average variance extracted (AVE) were used to test the convergent validity [48]. Table 3 shows that factor loadings exceed the minimum requirement that all loadings must be greater than 0.70. Composite reliability exceeds the minimum value (0.70) and the AVE for each construct also exceeds the minimum value (0.50) [50]. As a result, convergent validity is established.

Convergent validity measures whether items can effectively reflect their corresponding factor, whereas discriminant validity measures whether two constructs are statistically different [51].

In the final stage of measurement model analysis, Heterotrait—Monotrait Ratio (HTMT) criterion suggested by Henseler and Ringle was used to evaluate discriminant validity among constructs [52]. The HTMT scores of all the constructs do not violate the threshold value of 0.9, thus confirming the presence of discriminant validity across the constructs of interest (Table 4).

**Table 4:** Analysis of HTMT discriminant validity.

	CC	FL	PC	PI	PS
CC					
FL	0.760				
PC	0.742	0.517			
PI	0.809	0.829	0.543		
PS	0.833	0.853	0.734	0.669	

According to Hair et al. [48], multicollinearity describes how much the other factors in the analysis are correlated or can be used to explain a particular variable. Multicollinearity is a potential issue in the structural model and a variance inflation factor (VIF) value of 5 or above signifies the presence of the multicollinearity problem. The presence of multicollinearity in this study was checked using the value of the variance inflation factor (VIF). There is no problem with multicollinearity as the value of VIF is around three or below (Tables 5 and 6).

**Table 5:** Multicollinearity statistics (Outer Collinearity Statistics).

Item Codes	VIF
CC1	1.962
CC2	2.222
CC3	1.850
CC4	1.748
CC5	2.140
FL1	1.459
FL2	1.554
FL3	1.326
FL4	1.164
PC1	1.929
PC2	1.160
PC3	1.864
PS1	2.239
PS2	2.263
PS3	1.962
PI3	1.925
PI4	1.821

**Table 6:** Multicollinearity statistics (Inner Collinearity Statistics).

Item Codes	PC	PS	PI	FL	CC
PC				1.870	
PS				1.959	
PI				1.498	
FL					1.000
CC					

### Structural model

**Fig 2:**
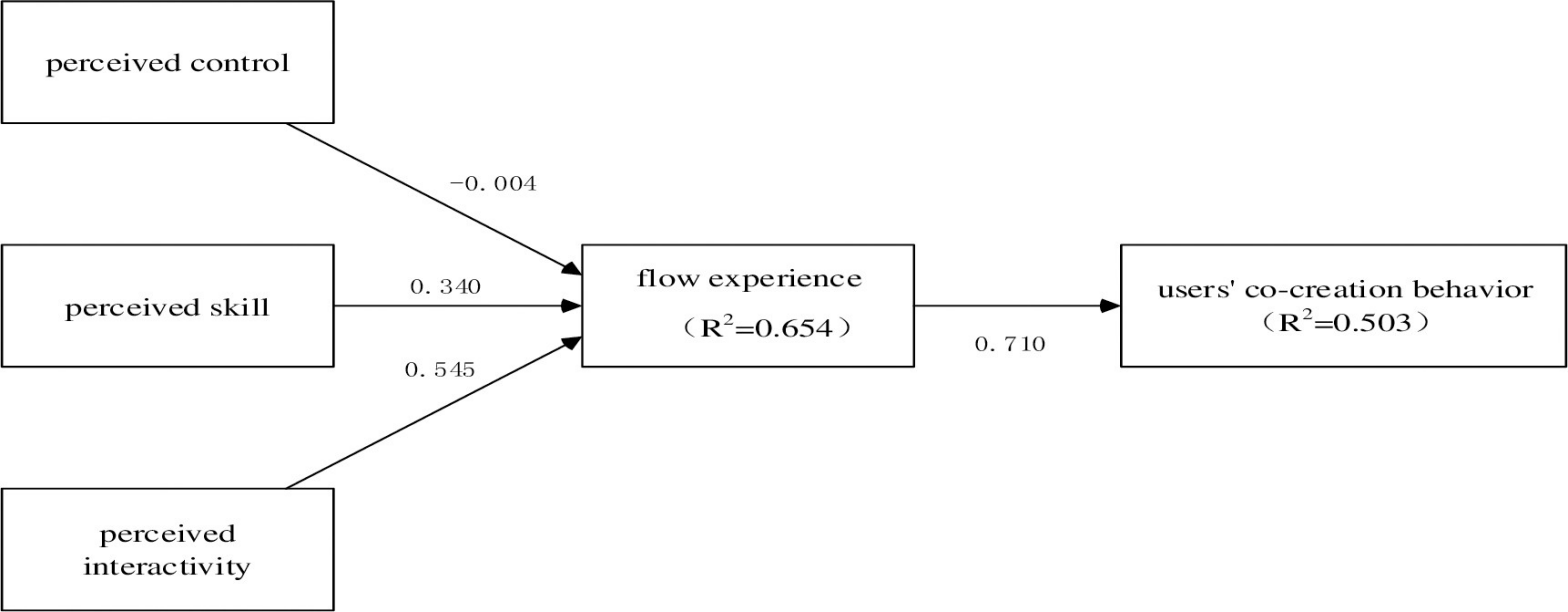
Structural model results (SEM).

The structural models for this research are presented below (Fig 2), in which R^2^ represents the value for any endogenous and predicted latent variable. R^2^ is 0.503 for the dependent variable, i.e. Co-creation. It means that Flow experience can explain 50.3 percent of the variance in Co-creation.

The bootstrapping option has been used to determine the statistical significance of the path coefficient and to calculate the t-values in this study (Table 7).

**Table 7:** Assessment of structural model with the bootstrapping procedure.

Path Hypotheses	Sample Mean	Std	T-value	P	Findings
H1) PC->FL	0.003	0.083	0.048	0.962	Unsupported
H2) PS->FL	0.335	0.094	3.618	< .001	Supported
H3) PI->FL	0.540	0.073	7.436	< .001	Supported
H4) FL->CC	O.710	0.056	12.654	< .001	Supported

The t-values of the hypothesized path of PC and FL is 0.048, which is below 2.57 (a = 0.01; two-sided test) and the p-value is 0.962. So, the hypotheses path of PC and FL of the inner model is not statistically significant.

The t-values of the hypothesized path of PS and FL is 3.618, which is above 2.57 (a = 0.01; two-sided test) and the p < .001. Therefore, the hypothesized path of PS and FL of the inner model is statistically significant.

The t-values of the hypothesized path of PI and FL is 7.436, which is above 2.57 (a = 0.01; two-sided test) and p < .001. Therefore, the hypothesized path of PS and FL of the inner model is statistically significant.

The last t-values of the hypothesized path of FL and CC is 12.654, which is above 2.57(a = 0.01; two-sided test) and p < .001. So, the hypothesized path of FL and CC of the inner model is statistically significant.

## Discussion and implications

### Discussion

Many researchers have discussed flow experience on social media to explore users’ behavior. This study adds an understanding of these behaviors in the context of music streaming platforms.

The result shows that perceived skill and perceived interactivity are statistically significant with the flow experience. And also the factor of flow experience is statistically significant with the co-creation behavior. This is consistent with past finding [53, 54]. There was no significant relationship between perceived control and flow experience, different from extant studies [26, 55], perhaps the users’ sense of control on music streaming platforms is not as strong as they are in other environments for there is increasing parity in the music streaming platforms. And due to the lack of perceived control, it is difficult to test the relationship between perceived control and flow experience. Therefore, for music streaming platforms, it would be easier to encourage co-creation behavior for those audiences who perceived skill and perceived interactivity than for those audiences who perceived control.

Overall, the findings of this study contribute to the extant literature by providing empirical evidence on users’ co-creation behavior on music streaming platforms. Therefore, the findings of this article have important implications for management policymakers, shareholders, and others who have an interest in users’ co-creation behavior on music streaming platforms.

### Implications

#### Theoretical implications

This study has two meaningful theoretical contributions. First, we extend the insights provided in past literature by demonstrating empirically that flow experience has an important effect on co-creation behavior on music streaming platforms. Flow is the holistic experience that people feel when they are involved in doing something [40]. They lose self-consciousness and ignore any other external stimulus only focusing on what matter. The existing literature has shown flow has a positive effect on social media users’ behavior, however, there has been lacking discussion regarding content co-creation on music streaming platforms. This study confirms the positive effect of flow experience on users’ co-creation behavior. Individuals who feel a sense of flow on music streaming platforms will have a positive attitude and will be more prone to content co-creating.

Second, we analyze the antecedents of flow experience on music streaming platforms and address two important variables in this context: perceived skill and perceived interactivity. Perceived skill is a key antecedent of flow experience because it is the user’s assessment of his or her capabilities to produce content and then express active behavioral intentions. We find that users who perceived high-level skills have more intention of co-creation than others whose perceived skill is at a low level. Thus users with a high level of perceived skill might focus on content co-creation on music streaming platforms because they believe in their capabilities to offer good ideas and information to improve the quality of content on music streaming platforms. Perceived interactivity is regarded as an antecedent of flow experience, for it is a strong stimulus creating a flow state in many behavioral reactions. Many studies demonstrated that perceived interactivity should be more discussed as an important factor that influences flow experience. This study showed that interactivity is a unique feature of music platforms that positively improves users’ flow. When users on music streaming platforms interact with each other, they will feel perceived interactivity, and then they learn more about music and increase their flow in enjoying music. This provides future researchers with insights to further explore the application of flow theory in other similar contexts.

#### Practical implications

This study also has some practical implications. First, managers of music platforms can stimulate customers’ flow by improving perceptions of interactivity. The music platform must take measures such as adding more relaxed and interesting topics, publishing attracting events, and inviting popular stars, musicians, composers, and other famous people to use the platform to increase interactivity among users. Managers of music platforms can also stimulate customers’ flow by improving perceptions of skill. They can create a spatial environment to make the users feel their competence in content creation and also supply some lectures or videos to introduce music knowledge to increase the users’ understanding of music to make sure enhance their perceived skill.

Second, this study implies that users’ flow is a desirable state that can occur while they use the platform. This suggests that managers of music platforms should strive to create a strategy to foster such an experience for their customers to trigger their motivation to co-create.

## Conclusion

The purpose of our study was to better discern the factors that affect the users’ co-creation behavior on music streaming platforms. The music industry has undergone profound changes due to the dematerialization of music and has been little studied. Music streaming platforms are characterized by strong interactions between producers and consumers through various consumers’ co-creation behavior.

We use the flow theory to identify how customers’ perceived control, perceived skill, and perceives interactivity influence their flow experience and then affect the co-creation behavior.

Our study also provides a set of theoretical and managerial implications resulting from the findings, which we expect to help future researchers and practitioners to enhance the customers’ co-creation intention on music streaming platforms, including recommendations related to how and why the platform should facilitate users’ flow for increasing the value co-creation behavior.

## Limitations and future directions

Our current study does also have some limitations. First, the respondents were voluntary participants, most of whom were college students. Even though this sample represents a fairly typical user on music streaming platforms, it does not represent all users. Future studies could test more samples to see if the results still hold.

Second, we investigated users’ co-creation behavior using a research framework based on flow theory. Future studies can investigate this issue using a broader variety of factors (social presence, social connectedness, affective commitment, etc.) and implement more theoretical models (SOR, UTAUT, online self-disclose, etc.)

Third, our study designed a cross-sectional test, but since users’ co-creation behavior is changeable when they use a music streaming platform, it cannot be easily confirmed whether flow experience affects their behavior over time, we can consider a longitudinal design in future research.
